# Prostate-Specific Membrane Antigen (PSMA) Promotes Angiogenesis of Glioblastoma Through Interacting With ITGB4 and Regulating NF-κB Signaling Pathway

**DOI:** 10.3389/fcell.2021.598377

**Published:** 2021-03-04

**Authors:** Yang Gao, Hui Zheng, Liangdong Li, Mingtao Feng, Xin Chen, Bin Hao, Zhongwei Lv, Xiaoyan Zhou, Yiqun Cao

**Affiliations:** ^1^Department of Neurosurgery, Fudan University Shanghai Cancer Center, Shanghai, China; ^2^Department of Oncology, Shanghai Medical College, Fudan University, Shanghai, China; ^3^Department of Nuclear Medicine, Shanghai Tenth People’s Hospital, Tongji University, Shanghai, China; ^4^Institute of Pathology, Fudan University Shanghai Cancer Center, Shanghai, China

**Keywords:** GBM, PSMA, angiogenesis, tumor progression, HUVEC

## Abstract

**Background:**

Glioblastoma multiforme (GBM) is the most common primary malignant tumor in the central nervous system (CNS), causing the extremely poor prognosis. Combining the role of angiogenesis in tumor progression and the role of prostate-specific membrane antigen (PSMA) in angiogenesis, this study aims to explore the functions of PSMA in GBM.

**Methods:**

Clinical GBM specimens were collected from 60 patients who accepted surgical treatment in Fudan University Shanghai Cancer Center between January 2018 and June 2019. Immunohistochemical staining was used to detect PSMA and CD31 expression in GBM tissues. Prognostic significance of PSMA was evaluated by bioinformatics. Human umbilical vein endothelial cells (HUVECs) transfected with PSMA overexpression plasmids or cultured with conditioned medium collected based on GBM cells, were used for CCK8, Transwell and tube formation assays. High-throughput sequencing and immunoprecipitation were used to explore the underlying mechanism. Furthermore, the *in vivo* experiment had been also conducted.

**Results:**

We demonstrated that PSMA was abundantly expressed in endothelium of vessels of GBM tissues but not in vessels of normal tissues, which was significantly correlated with poor prognosis. Overexpression of PSMA could promotes proliferation, invasion and tube formation ability of human umbilical vein endothelial cells (HUVECs). Moreover, U87 or U251 conditioned medium could upregulated PSMA expression and induce similar effects on phenotypes of HUVECs, all of which could be partially attenuated by 2-PMPA treatment. The mechanistic study revealed that PSMA might promote angiogenesis of GBM through interacting with Integrin β4 (ITGB4) and activating NF-κB signaling pathway. The *in vivo* growth of GBM could be alleviated by the treatment of 2-PMPA.

**Conclusion:**

This study identified PSMA as a critical regulator in angiogenesis and progression of GBM, which might be a promising therapeutic target for GBM treatment.

## Introduction

Glioma is the most common primary central nervous system (CNS) tumor, accounting for about 40–50% of intracranial tumors. Glioblastoma multiforme (GBM) is the most aggressive type of glioma in the CNS (Gusyatiner and Hegi, 2018). Despite comprehensive treatment including maximal surgical resection followed by adjuvant radiotherapy and chemotherapy, the median survival time of most patients is still less than 15 months, the 5 years survival rate is less than 3% (Bush et al., 2017; Reifenberger et al., 2017; Gusyatiner and Hegi, 2018). Thus, it is extremely urgent to explore novel therapeutic targets to improve the overall survival of glioma patients. Researchers have found that GBM is one of the most vascular-rich malignant tumors, and the aggressive nature of GBM is closely related with tumor angiogenesis (Touat et al., 2017). Thus, targeting the endothelium of GBM is attractive because endothelium can be exposed to an antibody much more readily than the tumor substance, which is protected by the blood-brain barrier.

At present, targeted therapy has become the most common treatment strategy against angiogenesis, which can inhibit the angiogenesis of tumors by targeting specific regulatory factors of tumor angiogenesis, thereby improving the prognosis of patients. Studies have found that GBM can express a variety of specific tumor angiogenesis regulators, and some of which have also been put into clinical use as targets for targeted therapy of GBM. For example, bevacizumab (targeting vascular endothelial growth factor [VEGF]) has been approved by FDA to be used for the treatment of GBM that is refractory or poorly responsive to other treatments (Diaz et al., 2017). However, the clinical application of current drugs has not yet significantly improved the prognosis of GBM patients due to the invasive growth of GBM and susceptibility to drug resistance. Therefore, the mechanistic study of tumor angiogenesis in GBM for exploring tumor-specific angiogenesis regulators as therapeutic targets is extremely important for GBM treatment.

In recent years, prostate-specific membrane antigen (PSMA) has attracted more and more attention in the field of tumor-related research. PSMA is highly expressed in prostate cancer epithelial cell membrane, whose expression is positively correlated with the growth and invasiveness of tumors. The application of radionuclide labeling of molecules with high affinity for PSMA in the diagnosis, staging, re-staging and treatment of refractory prostate cancer is increasingly becoming a new strategy for the imaging and treatment of prostate cancer. For example, ^68^Ga-PSMA PET/CT guided ^177^Lu-PSMA therapy has been considered as a promising direction for precise diagnosis and treatment of refractory prostate cancer (Maurer et al., 2016; Giovacchini et al., 2017). Notably, recent studies showed that, PSMA exhibits obvious expression in the neovascular structure of gliomas, especially GBM, but no expression in the blood vessels of normal brain tissue (Nomura et al., 2014; Evans et al., 2016; Sasikumar et al., 2018; Mahzouni and Shavakhi, 2019). However, to date, research concerning the relationship between PSMA and GBM was limited to descriptive observations by nuclear medical imaging or pathological staining. Herein, this study aimed to demonstrate the role and molecular mechanism of PSMA in GBM angiogenesis.

## Materials and Methods

### Clinical Specimens

GBM tissues and para-cancerous normal tissues were collected from 60 patients who received surgical treatment at Fudan University Shanghai Cancer Center between January 2018 and June 2019. Age, sex, and feature of MGMT, IDH1 or P53 mutations were also analysis. All patients were informed and informed consents were collected. Ethical approval was obtained from the ethics committee of the Fudan University Shanghai Cancer Center.

### Immunohistochemical Staining Assay (IHC)

Paraffin sections were deparaffinized in xylene followed by rehydration through graded alcohol and washed in PBS-T20. Then slides were immersed in EDTA antigen repair buffer (pH 9.0) and boiled for 30 min. After cooled in room temperature, slides were washed with PBS (pH 7.4). Next, all slides were blocked by 3% H_2_O_2_ for 25 min in dark, then incubated with 3% BSA or rabbit serum for 30 min. Primary antibodies CD31 (# GB13063, Proteintech 1: 200), PSMA (# ab19071, Abcam, 1:100) were applied at 4°C overnight. After washing, slides were incubated with streptomycin-HRP secondary antibody at room temperature for 1 h. Slides were colored with DAB and counterstained with 10% Harris hematoxylin for 3 min. Finally, the slides were dehydrated with gradient alcohol and sealed with neutral balsam. Pictures were collected under the Microscope (OLYMPUS) and analyzed with ImageJ. Slides incubated with 1% BSA were used as negative controls. The intensity of staining was scored as follows: faint, barely perceptible staining was scored 1; moderately intense staining was scored 2; and maximum-intensity staining was scored 3.

### Cell Culture

Cell lines including HUVECs, U87 and U251 were purchased from the cell library of the Chinese Academy of Sciences. All cell lines were grown in 1640-RPMI medium (Gibco) supplemented with 10% fetal bovine serum (FBS, Gibco) and incubated at 37°C in a humidified atmosphere with 5% CO_2_. The medium was refreshed every 3 days.

U87 and U251 cells were cultured in serum-free medium for 24 h, followed by the collection of the cell supernatant. Cells were cultured with U87 supernatant, U251 supernatant, 2-PMPA (100 μM, # M6154, AbMole) or normal medium, respectively, for 24 h, then cells were collected for following experiments.

### Construction of PSMA Overexpression Plasmids and Cell Transfection

Cells were seeded into 6 cm dishes. Next day, plasmids (10 μg) with PMSA overexpression sequence (the primers used for verification were listed below, 114 bp) or control plasmid was transfected into cells using Lipofectamine 2000 transfection reagent for 24 h culturing. Cells were harvested for downstream detection.

h-PSMA-F, 5′-CGGAGCAAACCTCGGAGTC-3′,
h-PSMA-R, 5′-GCGGCCAGAAACAATGGATAG-3′;
h-GAPDH-F, 5′-GGAGCGAGATCCCTCCAAAAT-3′,
h-GAPDH-R, 5′-GGCTGTTGTCATACTTCTCATGG-3′.

### RT-qPCR

Total RNA was extracted using TRIzol reagent (# 15596-018, Invitrogen), the quality and quantity was evaluated through NanoDrop 2000/2000C spectrophotometer (Thermo Fisher Scientific) according to the manufacturer’s instruction. RNA (2.0 μg) was reverse transcribed to cDNA using RNA cDNA first strand synthesis kit (# AT341, TransGen Biotech). Two steps qRT-PCR was performed with SG Fast qPCR Master Mix (# B639273, Sangon Biotech) and SYBRGreen Ex Taq^TM^ (# DRR100A, TAKARA) on ABI stepone plus fluorescence quantitative PCR instrument. The relative quantitative analysis in gene expression data was analyzed by 2^–ΔΔ*Ct*^ method. The primer sequences of PSMA and inner control GAPDH were as follows:

h-PSMA-F, 5′-CGGAGCAAACCTCGGAGTC-3′,
h-PSMA-R, 5′-GCGGCCAGAAACAATGGATAG-3′;
h-IL6-F, 5′-ACTCACCTCTTCAGAACGAATTG-3′,
h-IL6-R, 5′-CCATCTTTGGAAGGTTCAGGTTG-3′;
h-TNF-F, 5′-CTCGAACCCCGAGTGACAAG-3′,
h-TNF-R, 5′-TGAGGTACAGGCCCTCTGAT-3′;
h-CXCL1-F, 5′-CTGGCTTAGAACAAAGGGGCT-3′,
h-CXCL1-R, 5′-TAAAGGTAGCCCTTGTTTCCCC-3′;
h-EDN1-F, 5′-AGAGTGTGTCTACTTCTGCCA-3′,
h-EDN1-R, 5′-CTTCCAAGTCCATACGGAACAA-3′;
h-ICAM1-F, 5′-GTATGAACTGAGCAATGTGCAAG-3′,
h-ICAM1-R, 5′-GTTCCACCCGTTCTGGAGTC-3′;
h-GAPDH-F, 5′-GGAGCGAGATCCCTCCAAAAT-3′,
h-GAPDH-R, 5′-GGCTGTTGTCATACTTCTCATGG-3′.

### Cytotubule Formation Assay

Matrigel (# 356234, Corning) was dissolved and spread into the precooled 24-well plate (300 μL/well) and fixed for 30 min. 2 × 10^5^ HUVEC cells were seeded into each well and incubated at 37°C for 12 h. The tube-like structures were observed and pictured with a microscope.

### Cell Migration/Invasion Assay

Cell migration assay was performed in a 24-well plate (8 μm-pore) Transwell chamber inserts (Corning). 100 μL cell suspension was seeded into each well of the upper chamber, and 600 μL medium supplemented with 20% FBS was added into the lower chamber. After 24 h culturing, the Transwell chamber was taken out and washed. Cells migrated through the plate were fixed with formaldehyde (BBI Life Sciences) for 30 min, then stained with 0.1% crystal violet (BBI Life Sciences) for 30 min. Cells were imaged and counted under 400× light microscope (Olympus). On the other hand, transwell assay with Matrigel was used for the detection of cell invasion.

### Cell Counting Kit-8 Assay

Cell counting Kit-8 (CCK-8) (# E606335-0500, BBI Life Sciences) was used in cell proliferative detection. Briefly, cells were cultured in a 96-well plate (Corning) and 10 μL CCK-8 reagent was added into each well, and cultured at 37°C for 1 h without light. OD450 was measured via a Microplate Reader (Biotek).

### High Throughput Sequencing

PSMA negative control (NC) group and overexpress (OE) group were used for next generation sequencing. After RNA capturing, reverse transcribed to cDNA, amplification, and library generation, the next generation sequencing was performed. Statistical significance was assessed using Welch *t*-test with Benjamini-Hochberg FDR (|Fold Change| ≥ 2.0 and FDR < 0.05 as significant). Enrichment analysis of KEGG signaling pathway and GSEA analysis was executed with R software.

### Co-immunoprecipitation (Co-IP) Assay

Cells were lysed in RIPA buffer, and proteins were collected and determined by BCA protein assay kit (Beyotime Biotechnology). 40 μL protein sample with 10 μL precleared AgroseA/G Beads was incubated for 1 h at 4°C. Another 100 μL precleared AgroseA/G Beads was blocked with 10 μL BSA (50 mg/mL) for 1 at 4°C. Blocked beads were collected and resuspend with lysis buffer. The blocked beads (10 μL) were added into protein sample and incubated with 5 μL PMSA (1:10, # ab133579, Abcam), ITGB4 (1:250, # ab182120, Abcam) or rabbit non-specific IgG antibodies overnight. 1 × SDS and 5 × protein sample was added into collected beads and boiled for 10 min, then 40 μg samples were subjected to SDS-PVDF for separating. PVDF membrane was blocked with 5% skimmed milk TBST, and incubated with PSMA (1:50,000), ITGB4 (1:1,000) and GAPDH (1:5,000, # ab179467, Abcam) antibodies at 4°C overnight. Following incubated with secondary anti-mouse/ribbit IgG (HRP) (1:5,000, # 7076/7074, CST) for 1 h. Proteins were detected with enhanced chemiluminescence (Thermo Fisher Scientific) and blot images were analyzed using the ImageJ.

### Mice Xenograft Model

Nude mice were housed at pathogen-free condition, and divided into two groups (Ctrl Group and 2-PMPA Group) randomly before experiments. U87-luc cells were intracranially injected into mice. After the formation of tumors, a treatment of 2-PMPA (50 mg/kg body weight) was carried out in the next 2 weeks. For the *in vivo* imaging, 150 mg/kg luciferin (as a 15 mg/mL solution in DPBS) was injected, and followed by anesthesia of mice by isoflurane. The mice were placed in the imaging system to detect tumor cells. The exposure time ranged from 1 min to 1 s. The imaging results were expressed in photons per second through the processing of imaging software. The biological imaging was performed once before the 2-PMPA administration, and again after 2 weeks of 2-PMPA treatment. Ethical approval was obtained from the ethics committee of the Fudan University Shanghai Cancer Center.

### Western Blot

Xenografts were lysed in lysate containing (RIPA buffer + PMSF + phosphatase inhibitor), and proteins were collected and determined by BCA protein assay kit (Beyotime Biotechnology). Protein samples (10 μg) were separated by 10% SDS-PAGE gel, and then transferred to a poly(vinylidene fluoride) (PVDF) membrane (Millipore). PVDF membrane was blocked with 5% skimmed milk TBST at room temperature for 40 min, and incubated with PSMA (1:1,000), ITGB4 (1:1,000), p-P65 (1:1,000, # 3033, CST), P65 (1:1,000, # ab32536, Abcam) and GAPDH (1:5,000, # ab179467, Abcam) antibodies at 4°C overnight. Following incubated with secondary anti-mouse/ribbit IgG (HRP) (1:5,000, # 7076/7074, CST) for 1 h. Proteins were detected with enhanced chemiluminescence (Thermo Fisher Scientific) and blot images were analyzed using the ImageJ.

### Statistical Analysis

Each experiment in this research was performed independently at least three times and data was expressed as Mean ± *SD*. The differences between groups were determined with Student’s *t*-test or one-way ANOVA test. All analyses were performed on IBM SPSS 21.0 Software and GraphPad Prism 8.0 Software. *P*-values less than 0.05 were considered statistically significant.

## Results

### PSMA Is Abundantly Expressed in Neovascular Structure of GBM and Predicts Poor Prognosis

**FIGURE 1:**
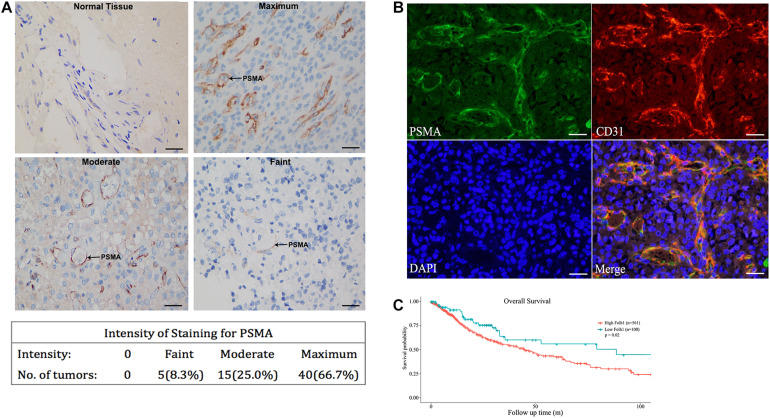
PSMA is abundantly expressed in neurovascular structure of GBM and predicts poor prognosis. **(A)** IHC was performed to detect PSMA expression in normal and GBM tissues and representative images of GBM tissues with faint, moderate and maximum PSMA expression were shown (Scale bar = 100 μm). **(B)** The Immunofluorescence colocalization detection of CD31 and PSMA in GBM tissues (Scale bar = 100 μm). **(C)** Kaplan-Meier survival analysis was performed to reveal the significance of PSMA expression in prognosis of GBM patients.

This study included 38 males and 22 females, with a media age of 49 years (range from 30 to 72 years). There was no significant correlation between the expression of PSMA and MGMT, IDH1, and P53 mutations. In order to explore the role of PSMA in angiogenesis of GBM, its expression was first detected in GBM tissues and normal brain tissues by immunohistochemical staining. As shown in Figure 1A, no PSMA expression could be observed in vessels of normal brain tissues, while PSMA was faintly expressed in endothelium of vessels in 5/60 GBM tissues, moderately expressed in 15/60 GBM tissues and highly expressed in 40/60 GBM tissues (Figure 1A). Moreover, in order to verify the localization of PSMA expression in GBM, a vascular endothelial marker CD31 staining was performed to visualize the neovascular structure of GBM. The results demonstrated that the expression level and localization of PSMA in GBM tissue was consistent with CD31, indicating the potential role of PSMA in angiogenesis of GBM (Figure 1B). Furthermore, through collecting the data of PSMA expression and patients’ prognosis from The Cancer Genome Atlas (TCGA) database, a Kaplan-Meier survival analysis was conducted to show that GBM patients with relatively higher PSMA expression suffered from poorer prognosis (Figure 1C).

### Overexpression of PSMA Promotes Proliferation, Invasion and Tube Formation of HUVECs

**FIGURE 2:**
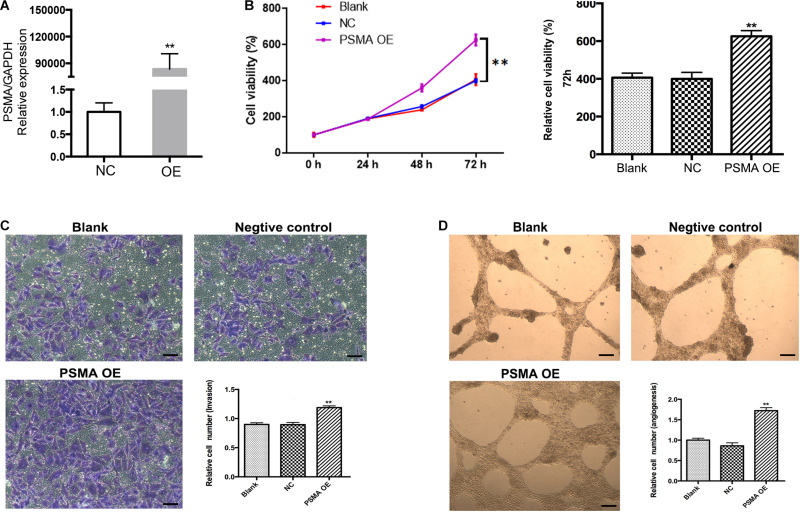
Overexpression of PSMA promotes proliferation, invasion and tube formation of HUVECs. **(A)** qPCR was used to detect PSMA expression in HUVECs with or without PSMA overexpression. **(B)** CCK8 assay was performed to examine the effects of PSMA overexpression on proliferation of HUVECs. **(C)** Transwell assay was used to evaluate the effects of PSMA overexpression on invasion ability of HUVECs (Scale bar = 100 μm). **(D)** Tube forming assay was carried up to assess the effects of PSMA overexpression on tube formation ability of HUVECs (Scale bar = 50 μm). Data were presented as mean with *SD*. **P < 0.01.

In order to investigate whether PSMA could promote tumor angiogenesis, cell model based on human HUVECs transfected with PSMA overexpression plasmids was constructed. As detected by qPCR, PSMA was overexpressed by approximate 90,000-fold in OE group compared with that in the NC group (Figure 2A). Subsequently, the CCK8 and Transwell assays were performed to evaluate the effects of PSMA overexpression on proliferation and invasion of HUVECs, respectively. The results declared that PSMA overexpression significantly promoted the proliferation and invasion ability of HUVECs (Figures 2B,C). More importantly, PSMA overexpression could improve the tube formation ability of HUVECs (Figure 2D). All these results suggested that PSMA might play an important role in the biological functions of HUVECs.

### PSMA Plays Critical Role in Angiogenesis of GBM

**FIGURE 3:**
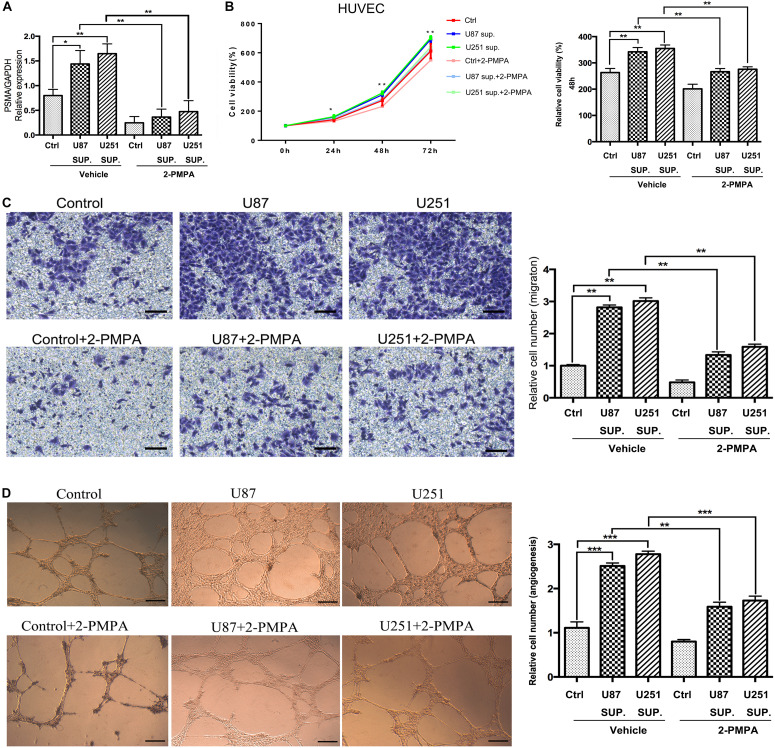
PSMA plays critical role in angiogenesis of GBM. **(A)** qPCR was used to detect PSMA expression in HUVECs cultured with normal medium or U87/U251 conditioned medium, and treated with vehicle or 2-PMPA. **(B)** CCK8 assay was performed to examine the effects of U87/U251 conditioned medium culturing and inhibitor 2-PMPA on proliferation of HUVECs. **(C)** Transwell assay was used to evaluate the effects of U87/U251 conditioned medium culturing and inhibitor 2-PMPA on invasion ability of HUVECs (Scale bar = 100 μm). **(D)** Tube forming assay was carried up to assess the effects of U87/U251 conditioned medium culturing and inhibitor 2-PMPA on tube formation ability of HUVECs (Scale bar = 50 μm). Data were presented as mean with *SD*. *P < 0.05, **P < 0.01, ***P < 0.001.

Given the high expression of PSMA in GBM and its functions in promoting proliferation, invasion and tube formation ability of HUVECs, role of PSMA in GBM angiogenesis was investigated through culturing HUVECs with conditioned medium collected from U87 and U251 cell lines. As shown in Figure 3A, HUVECs cultured with U87 or U251 conditioned medium showed significantly higher PSMA expression compared with that cultured with normal medium EBM-2. Meanwhile, the treatment of PSMA inhibitor 2-PMPA could effectively eliminate PSMA expression in all groups (Figure 3A). The following cell phenotypes detection showed that U87 or U251 conditioned medium could significantly accelerate proliferation, promote migration and enhance tube formation ability of HUVECs, all of which could be partially attenuated by 2-PMPA treatment (Figures 3B–D). Altogether, these results indicated the essential role of PSMA in tumor angiogenesis of GBM.

### PSMA Promotes GBM Angiogenesis Through Regulating NF-κB Signaling Pathway

**FIGURE 4:**
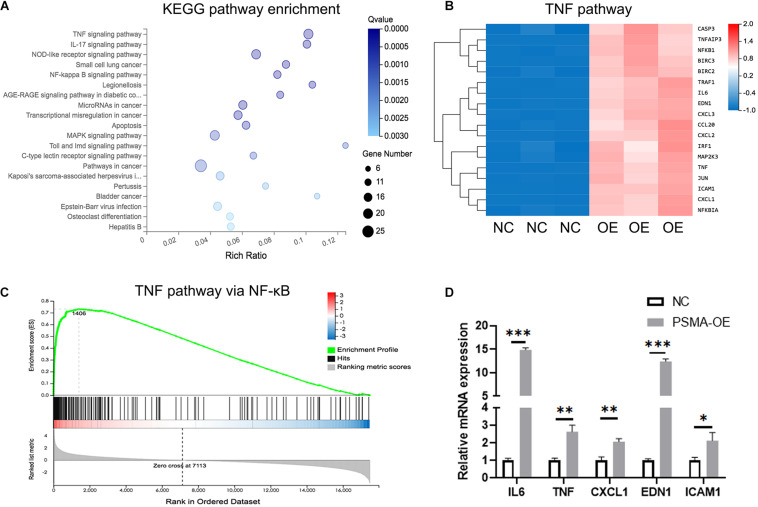
PSMA promotes GBM angiogenesis through regulating NF-κB signaling pathway. **(A)** The enrichment analysis of DEGs identified by RNA sequencing was performed based on KEGG. **(B)** The heat-map showed the expression of DEGs of TNF signaling pathway in HUVECs with or without PSMA overexpression. **(C)** GSEA analysis indicated that DEGs in TNF signaling pathway were significantly enriched in NF-κB signaling pathway. **(D)** Five key factors in TNF or NF-κB signaling pathways, including IL-6, TNF-α, CXCL1, EDN1 and ICAM1 were subjected to qPCR for detecting their expression in HUVECs with or without PSMA overexpression. Data were presented as mean with *SD*. *P < 0.05, **P < 0.01, ***P < 0.001.

In order to further explore the mechanism underlying the regulatory effects of PSMA on GBM angiogenesis, an RNA sequencing was performed to identify differentially expressed genes (DEGs) between NC group (*n* = 3) and OE group (*n* = 3) of HUVECs. Based on all the DEGs, an enrichment analysis of KEGG signaling pathway showed TNF signaling pathway as the most enriched pathway (Figure 4A). It was shown that 18 signal transduction factors were significantly upregulated in HUVECs with PSMA overexpression (Figure 4B). Moreover, further gene-set enrichment analysis (GSEA) indicated that DEGs in TNF signaling pathway were significantly enriched in NF-κB signaling pathway, which was a well-known downstream of TNF signaling pathway (Figure 4C). Finally, factors including IL-6, TNF, CXCL1, EDN1, and ICAM1 were selected for further verification by qPCR, all of which were upregulated in PSMA overexpressed HUVECs (Figure 4D). Herein, all the results suggested that PSMA might regulate biological functions of HUVECs through activating NF-κB pathway.

### PSMA Promotes GBM Angiogenesis Through Interacting With ITGB4

**FIGURE 5:**
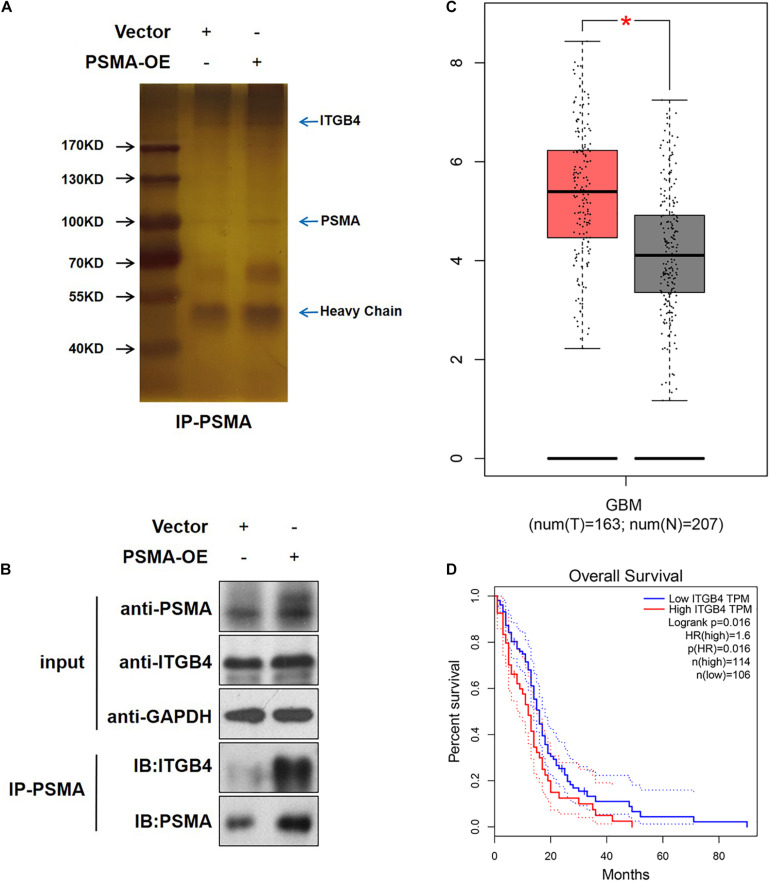
PSMA promotes GBM angiogenesis through interacting with ITGB4. **(A)** HUVECs with or without PSMA overexpression were lysed and immunoprecipitated with anti-PSMA antibody. **(B)** The expression of PSMA and ITGB4 was detected in input complex, and the expression of PSMA was detected in IP complex. **(C)** The expression of ITGB4 in GBM tissues (left, *n* = 163) and normal tissues (right, *n* = 207) was analyzed through GEPIA online tools. **(D)** The correlation between ITGB4 expression and GBM patients’ prognosis was analyzed through GEPIA online tools. *P < 0.05.

An IP assay was performed to identify PSMA-interacting proteins (Figure 5A). The results demonstrated that ITGB4, an activator of NF-κB signaling pathway, was proved to be directly interaction with PSMA (Figure 5B). Therefore, it could be deduced that PSMA might promote GBM angiogenesis through interacting with ITGB4. Furthermore, we explored the role of ITGB4 in GBM using web resource GEPIA, of which the data originated from the TCGA and GTEx. The results showed that ITGB4 was significantly upregulated in GBM tissues in comparison (Figure 5C) with normal tissues and also predicted poor prognosis (Figure 5D).

### PSMA Promotes the Development of GBM *in vivo*

**FIGURE 6:**
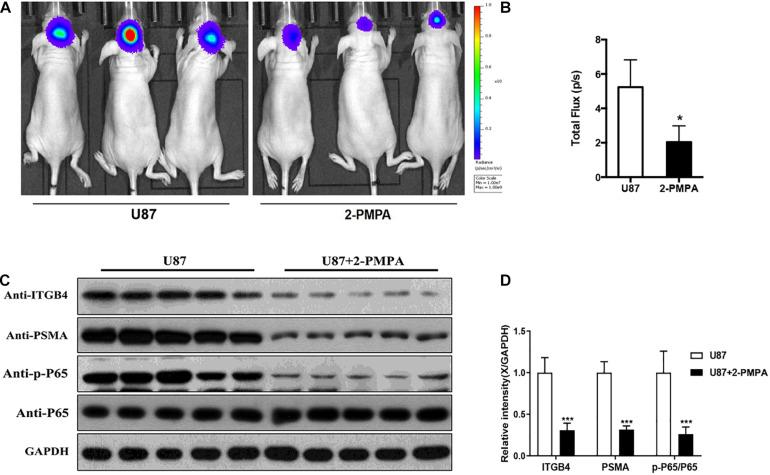
PSMA promotes GBM development *in vivo*. **(A)** Bioluminescence imaging was performed to evaluate the growth of xenografts, **(B)** and the intensity of which was scanned as a representation of tumor growth. **(C,D)** The expression of PSMA, ITGB4, and phosphorylation of NF-κB p65 were also detected after using inhibitor 2-PMPA *in vivo*. Data were presented as mean with *SD*. *P < 0.05.

In order to further demonstrate the functions of PSMA in the development of glioma, orthotopic xenograft models were constructed through the intracranial injection of U87-luc cells and divided into Ctrl group and 2-PMPA group, mice in which were treated by 2-PMPA for 2 weeks after the formation of xenografts. Bioluminescence imaging was performed after the injection of D-luciferin and used for evaluating the growth of tumors. As shown in Figures 6A,B, the administration of 2-PMPA could significantly suppress the *in vivo* growth of GBM, suggesting the important role of PSMA in GBM development. Moreover, we also observed the downregulation of PSMA, ITGB4 and phosphorylation of NF-κB p65, which was in agreement with the abovementioned results (Figures 6C,D).

## Discussion

PSMA is a unique complete type II prostate epithelial cell glycoprotein that spans the cell membrane and consists of 19 intracellular amino acids, 24 transmembrane amino acids and 707 extracellular amino acids. The overexpression of PSMA in prostate cancer has been well documented, which has been widely applied, as a molecular target, in the clinical diagnosis and treatment of prostate cancer and exhibited extremely high sensitivity and performance. On the other hand, PSMA is also found to express in neovascular structures or endothelial cells of various types of solid tumors, suggesting its potential relevance to tumor angiogenesis. For example, Haffner et al. (2009) reported the expression of PSMA in the neovascular system of gastric cancer and colorectal cancer. Similarly, several studies discovered the expression of PSMA in neovascularization of breast cancer and thyroid tumors through immunohistochemical staining and molecular imaging of nuclear medicine (Haffner et al., 2009; Alla Gabriella et al., 2014; Derlin et al., 2017). Conway et al. (2006) made a preliminary exploration of the role and mechanism of PSMA in angiogenesis. The results showed that PSMA could enhance the aggressiveness of HUVEC cells by promoting the activities of integrin β1 and PAK (p21-activated kinase)-1, thereby affecting angiogenesis. The activation of PAK-1 could interference the interaction between PSMA cytoplasmic tail region and anchor protein filamin A to reduce the activity of PSMA, while the inhibition of Integrin β1 activity can also reduce the activity of PSMA. Therefore, PSMA, integrin β1 and PAK-1 formed a self-regulated regulatory cycle and participated in angiogenesis. Liu et al. (2011) and Nguyen et al. (2016) further demonstrated that the conditioned medium obtained from kidney cancer, colorectal cancer, breast cancer, prostate cancer, ovarian cancer, and lung cancer cells can promote the expression of PSMA in HUVECs and enhance tube formation ability. All these results suggested the important role of PSMA in tumor angiogenesis. More specifically, some characteristics of PSMA, such as the relatively low expression in normal tissues, its localization on cell membrane and rapid internalization, make it a fairly promising candidate as target of anti-tumor drugs (Wernicke et al., 2011; Nomura et al., 2014).

As mentioned above, accumulating evidence showed that PSMA expression could be observed in neovascularization of GBM tissues, but not normal brain tissues (Evans et al., 2016; Sasikumar et al., 2018; Mahzouni and Shavakhi, 2019). For example, the work of Saffar et al. (2018) detected the expression of PSMA in Grade I ∼ IV GBM tissues by immunohistochemical staining. The results revealed that the expression level of PSMA in GBM tissues showed high consistency with that of neovascular marker CD31. In other words, higher PSMA and CD31 expression could be observed in GBM tissues with higher grade (Saffar et al., 2018). A number of recent studies in nuclear medicine imaging not only confirmed the high expression of PSMA in GBM relative to normal brain tissues or low-grade GBM tissues, but also showed that, through ^68^Ga-PSMA or PSMA antibody loaded ^89^Zn- Df-IAB2M, the PET/CT imaging possessed the ability to visualize the neovascularization of GBM and guide the targeted therapy of GBM (Schwenck et al., 2015; Kunikowska et al., 2018; Matsuda et al., 2018). All the studies showed that the specific expression of PSMA in neovascularization of GBM make it a promising therapeutic target for angiogenesis related therapy.

In this study, we demonstrated that PSMA was abundantly expressed in endothelium of vessels of GBM tissues but not in vessels of normal tissues, which was significantly correlated with poor prognosis. The upregulation of PSMA expression induced by lentivirus-mediated PSMA overexpression or culturing with U87/U251 conditioned medium could promote proliferation, migration, invasion and tube formation ability of HUVECs, which could be alleviated by PSMA inhibitor 2-PMPA. Furthermore, we also observed that the *in vivo* growth of GBM could be alleviated by the treatment of 2-PMPA. All these results indicated the potential of PSMA in the tumor angiogenesis of GBM.

In this study, through RNA sequencing and enrichment analysis of KEGG signaling pathway, we identified that PSMA may regulate phenotype of HUVECs through activating NF-κB signaling pathway, which was further proved by the downregulated expression of p-P65 in xenografts treated with PSMA inhibitor. NF-κB is a multifunctional transcription factor, which is activated by TNF-α, growth factors, cytokines, etc., thus participating in the regulation of various cellular functions such as proliferation, apoptosis, invasion, and migration. Moreover, studies have manifested that abnormal activation of the NF-κB signaling pathway can cause an increase in the concentration of VEGF in the tumor microenvironment, thereby promoting tumor neovascularization and causing invasion of cancer cells (Zanca et al., 2017). For example, Liang et al. (2017) reported that the upregulation of tumor suppressor GPER could inhibit the angiogenesis of triple negative breast cancer through de-activating NF-κB/IL-6 axis. Moreover, Jiang et al. (2013) indicated that the NF-κB signaling pathway, as the downstream mechanism of Bmi-1, mediated the Bmi-1 induced tumor angiogenesis of GBM. On the other hand, through protein identification by mass spectrum following PSMA-related immunoprecipitation, we discovered the direct interaction between PSMA and ITGB4, suggesting that ITGB4 may be the downstream target of PSMA to regulate tumor angiogenesis of GBM. Consistently, results of bioinformatics analysis indicated the upregulation of ITGB4 in GBM and the correlation between ITGB4 high expression and poor prognosis. ITGB4, also known as integrin β4, could regulate apoptosis, migration, invasion, and signal transduction of cancer cells through forming α6β4 with Integrin α6 (Niu and Li, 2017; Koivisto et al., 2018). In addition, accumulating evidence showed that ITGB4 was involved in the regulation of cell death, senescence and differentiation of other types of cells such as HUVECs (Sun et al., 2010; Wang et al., 2012; Siddharth et al., 2018). More importantly, ITGB4 was also reported as an activator of NF-κB signaling pathway to promote epidermal growth and wound healing (Nikolopoulos et al., 2005; Bhatia et al., 2013).

It has been well acknowledged that PSMA is a highly specific regulator of angiogenesis in a wide range of malignant tumors including GBM. Besides the observation of PSMA in neovascularization of GBM tissues other than normal tissues, we further confirm the GBM-induced PSMA upregulation and PSMA-induced enhancement in cell proliferation, migration, invasion and tube formation ability of HUVECs. Furthermore, it was also demonstrated that PSMA probably regulate phenotypes of HUVECs through interacting ITGB4 and activating NF-κB signaling pathway in GBM. Although this study still has some drawbacks such as the limited number of clinical specimens and plain mechanism research, all the characteristics and functions of PSMA make it a fairly promising therapeutic target for anti-angiogenesis therapy of GBM.

## Data Availability Statement

The raw data supporting the conclusions of this article will be made available by the authors, without undue reservation.

## Ethics Statement

The studies involving human participants were reviewed and approved by the ethics committee of the Fudan University Shanghai Cancer Center. The patients/participants provided their written informed consent to participate in this study. The animal study was reviewed and approved by Ethical approval was obtained from the ethics committee of the Fudan University Shanghai Cancer Center.

## Author Contributions

YG and LL designed this program. MF and XC operated the cell and animal experiments. BH conducted the data collection and analysis. YG and HZ produced the manuscript, which was checked by YC. XZ and ZL revised the manuscript. All authors have confirmed the submission of this manuscript.

## Conflict of Interest

The authors declare that the research was conducted in the absence of any commercial or financial relationships that could be construed as a potential conflict of interest.
